# Transplanting the Elderly: Mandatory Age- and Minimal Histocompatibility Matching

**DOI:** 10.3389/fimmu.2020.00359

**Published:** 2020-03-12

**Authors:** Geertje J. Dreyer, Johan W. de Fijter

**Affiliations:** Department of Internal Medicine (Nephrology), Leiden University Medical Center, Leiden, Netherlands

**Keywords:** kidney transplantation, elderly, allocation, histocompatibility, HLA, old-for-old program

## Abstract

Worldwide over 40% of patients receiving renal replacement therapy (RRT) are aged 65 years or older, a number that is still increasing. Renal transplantation is the preferred RRT, providing substantial survival benefit over those remaining on dialysis, including the elderly. Only 3% of patients aged 65 years or older accepted on the waiting list actually received a kidney transplant offer within the Eurotransplant allocation region. To increase the chance for elderly to receive a timely kidney transplant, the Eurotransplant Senior Program was introduced. The ESP supports local allocation of older kidneys to older donors in order to decrease cold ischemia time, while disregarding former exchange principles based on matching for HLA antigens. As a consequence, more elderly received a kidney transplant and a relative higher incidence of acute rejection resulted in additional courses of high steroids and/or depleting antibody therapy. Since death with a functioning graft due to infections is the dominant reason of graft loss in elderly, more intense clinical immunosuppression to prevent or treat acute rejection is not a very attractive option. Therefore in elderly kidney transplant candidates, we advocate reintroduction of minimal histocompatibility criteria (i.e., HLA-DR matching) followed by age-matching with mandatory local/regional allocation to also facilitate short cold ischemia.

## Introduction

End stage renal disease (ESRD) is a rapidly becoming a critical problem worldwide. In The Netherlands, for instance, the prevalence of patients receiving renal replacement therapy (RRT) was 17531 or 1020 per million population (pmp) in the year 2017, an increase of almost 35% in the last decade (1). In countries contributing to the ERA-EDTA registry the prevalence of RRT was 592779 (854 pmp) in 2012, a number that also increased almost 30% in recent annual reports (2). In the US even greater numbers have been documented with a prevalence of 726331 patients (2206 pmp) on RRT in 2016, a number that almost doubled since 2000 [(3) USRDS annual data report: Epidemiology of kidney disease in the United States National Institutes of Health]. With increasing numbers of senior citizens and associated health care challenges, even more elderly with chronic kidney disease and need for RRT can be anticipated. Nowadays, according to the Dutch National Renal Replacement Database (Renine) and the ERA-EDTA registry, already 45% of patients on RRT are 65 years of age or older (2). Likewise, in the US 41% of the patients with ESRD is over the age of 65 years.

It is known that patients aged 65 years or older, and especially those over 75 year, constitute a separate group with different views and needs regarding health care issues. This is also reflected in choice of RRT. Although kidney transplantation is generally the preferred treatment option when it comes to survival benefit across all ages (4–7), only 3% of the patients between 65 and 74 years were actually transplanted in 2017 and virtually none of the patients receiving a kidney transplant were over the age of 75 years (2). The majority of older transplant candidates are likely to die while on the waiting list before they get a transplant offer according to the Dutch Renine data. Mortality early after transplantation is also higher in elderly with a large French registry study reporting a 3-fold higher mortality risk in the first 3 postoperative months as compared to waitlisted counterparts (8).

Another important cause of death after kidney transplantation is failure of the kidney allograft, which is an important independent risk factor of mortality. After graft loss, the risk of mortality in those relisted for a repeat transplant is also higher as compared to patients with a functioning graft or those listed for their first transplant (9–11). Since all-cause mortality increases with age, the longevity of the first kidney graft, even allowing less optimal renal function, is of critical importance.

In this paper we reconsider the relative importance and causes of graft failure in the elderly as well as the challenges, hurdles and potential different approaches to prolong survival. We focus on the elderly and need for carefully balanced strategies in this vulnerable group of patients with ESRD.

## The Elderly With a Failing Kidney Transplant

Overall, kidney graft survival improved significantly in the past decades and mainly due to the prevention of early acute rejection by the use of more potent immunosuppressive drugs like tacrolimus, mycophenolate and more frequent use of poly- or monoclonal antibodies as induction therapy. The improvement in short-term and long-term graft survival has not improved concomitantly and long-term graft survival more or less stabilized between 1988 and 2005 (12). This trend continued beyond 2005 with an approximate 5% annual loss of kidney allografts after the 1st year (13). An analysis of the Collaborative Transplant Study (CTS) registry confirmed that also in Europe graft survival has improved mainly due to short term outcome parameters. Since the year 2000, however, there has been a lack of improvement in short- and long-term graft survival, even after taking into account the changing donor and recipient demographics and donor or organ quality characteristics. This observation probably reflects a lack of further innovation in the management and treatment options around the kidney transplant procedure (14).

Older kidney transplant recipients probably require different allocation and/or treatment strategies as compared to their younger counterparts. A critical first consideration is the notion that the elderly that actually do get a transplant offer are a highly selected subgroup of elderly patients with ESRD. First, these patients are generally rigorously medically selected before acceptance on the active kidney waiting list. Secondly with increasing age, only very low proportions of these patients actually reach the end of the cue, while significant numbers of patients are removed from the list due to comorbidities and the majority die while waiting for a kidney transplant offer. Taken together, there is an important selection based on cardiovascular and/or oncological exclusion criteria. Where these issues are important long-term topics for younger transplant recipients, in elderly transplant candidates or recipients these are not the main reason for graft loss and therefore may require other strategies.

### The Elderly and Mortality

In elderly the most important reason for late allograft loss is death with a functioning graft (DWFG) (15, 16). In a large registry using UNOS and USRDS data, Ojo et al. (17) found that 42,5% of graft loss was due to DWFG. Age at transplantation was an obvious, strong and independent risk factor. When compared to those aged 18–29 years, recipients aged over 65 years had a 7-fold increased risk to die with a functioning graft. Besides age, ESRD caused by systemic vascular diseases such as hypertension or diabetes mellitus was an independent risk factor for premature death (17). This was confirmed in a study of El-Zoghby et al. (18) where 43.4% of the grafts were lost due to death. Interestingly, the most important cause of death more than 5 years after transplantation was due to infectious causes. Transplant recipients who died due to infections were older (64.6 vs. 59.4 years) as compared to those due to cardiovascular diseases. This observation supports the fact that elderly kidney transplant recipients are an already highly selected group with excess infectious comorbidity and mortality, the downside of current potent clinical immunosuppressive drugs, and therefore a key consideration comparable to the increased cardiovascular risk in younger recipients (18).

Death due to infectious causes is the consequence of clinical overimmunosuppression, especially in elderly with an already immunosenescent immune system in the context of aging (19). In the light of solid organ transplantation, the consequence of the aging immune system has been documented in a shift from naïve T-cells toward relatively more memory T-cells resulting in decreased immune reactivity (20–22). Indeed the incidence and severity of infections parallels the increase in age in patients (23). Regarding renal transplant recipients, several studies have reported that older patients have more infectious problems and that older recipients die more often due to infectious causes (24, 25). Recently, Lemoine et al. (16) studied renal recipients over 70 years of age and confirmed the mortality risk in elderly due to infections. In a total of 171 recipients death-censored 1-year graft survival was 82.6 and 9.9% of included patients died after the 1st year with infectious causes in 58.5% of cases (16).

### Kidney Transplant Failure

#### The Elderly and Rejection

It has become widely accepted that older transplant recipients may encounter less acute rejection episodes after transplantation as compared to younger recipients due to immunosenescence (26, 27). However, if they do experience acute rejection, this episode is more likely to compromise graft- and/or patient survival, the latter in particular due to additional excessive immunosuppression (28). In a large registry, 5-year death-censored graft survival after rejection was 59.9% in recipients aged 65 years or older as compared to 82.1% in recipients aged 18 to 35 years (29).

Overall the frequency of rejection declines within subsequent age categories, but a higher donor age is significantly associated with higher rejection rates (30–32). A study in 2016 among 244 elderly also showed that although older recipient age was protective for the occurrence of acute rejection, this was clearly outweighed by the dominant negative effect of donor age and increased immunogenicity of the organ reflected by more rejection and more donor specific antibody (DSA) formation with increased HLA-DR mismatch (33). This study shows that 1 or 2 HLA-DR mismatches give a higher chance on TCMR and the development of DSAs which both results in decreased allograft survival (66% for TCMR and 63% for dnDSA compared to 82 and 80% resp). They also show a graded effect, patients with 2 HLA-DR mismatches had worse graft survival rates after 3 and 7 years after transplantation compared to 0 or 1 HLA-DR mismatch (80%, 76% and 73% for 0, 1 and 2 HLA-DR mismatches 7 years after transplantation). This complex interaction of risks for increased rejection incidences was confirmed by a Dutch group in 2017 where elderly recipients with an older DCD (donation after circulatory death) kidney had a 2.78 times higher risk of delayed graft function and rejection compared to elderly receiving a young DBD kidney (34). This increased immunogenicity in recipients of a more vulnerable kidney allograft could be due to more endothelial activation in the context of ischemia-reperfusion injury, bacterial and viral infections resulting in a more pro-inflammatory cytokine environment, increased expression of HLA molecules and/or recruitment of antigen-presenting cells (35). These data are especially relevant regarding renal transplantation in elderly, since expansion of the donor pool with older, high-risk kidney donors, is a key strategic policy for this subgroup of renal recipients. Especially in view of rejection treatment and higher risk on infections cumulating in the most important cause of death in elderly. Therefore, the optimal strategy to decrease rejection risks while still allowing a timely transplant in the elderly is of critical importance.

#### Donor Specific Antibodies

When addressing transplant failure, *de novo* donor specific antibodies against HLA antigens (dnDSAs) after transplantation gained more and more interest. Overall Lachmann et al. (36) reported significant a lower 10-year graft survival being 49% versus 83% in patient with and those without DSA, respectively. A recent study showed that DSAs in combination with other risk factors can be even more detrimental for graft function. In this study, DSAs were associated with an increased incidence of T cell mediated rejection (TCMR) and led to a three-fold increase in graft loss (37). Lemoine et al. (16) showed that anti HLA antibodies are an independent risk factor for patient death and graft loss within the 1st year in patients older than 70 years. In elderly their role was recently debated by von Moos et al. (38) since elderly have a lower risk in developing DSAs than pediatric patients. However, they found more dnDSA in patients treated with cyclosporine as compared to tacrolimus so regarding immunosuppressive protocols for elderly, their role is still important in long term graft survival.

Multiple studies have been performed to address the prevalence, risk factors and consequences of dnDSA. Most studies report a prevalence of dnDSA of 10–19% after kidney transplantation and most are formed in the 1st year after transplantation with an annual incidence of 5% thereafter (39–43). There are several risk factors for the formation of DSA and not surprisingly, non-adherence or lowering immunosuppressive drugs for clinical reasons play a crucial role (44–48). However, one can only form antibodies if there is a foreign HLA molecule and therefore the main risk factor is the degree of HLA mismatch between the recipient and the donor (49). Several studies show that HLA class II mismatch, in particular HLA-DQ, is most important (40, 41, 50). Other described risk factors for the formation of dnDSA are kidneys of deceased donors and younger age of the recipient.

Despite the current knowledge there is still no clearly defined clinical advice regarding DSAs and the prevention of formation. Guidelines from the Transplantation Society, the sensitization in transplantation: assessment of risk (STAR) working group and the Heidelberg algorithm, based on the CTS and data from the Heidelberg Transplant Center, all advise to test post-transplantation in pre-specified patient groups. All agree that patients most at risk are patients with a pre-activated immune system, measured by pre-existing antibodies or soluble CD30, in combination with periods of under-immunosuppression and should be monitored closely (51–53).

HLA compatibility between donor and recipient is currently assessed by the number of HLA mismatches on serologic level although HLA antibodies recognize accessible polymorphic sequences of amino acids rather than whole HLA antigens. These polymorphic sequences, so called epitopes, can be shared between HLA antigens so the true mismatch is much more complicated than serologic level shows. Therefore, the question can be raised whether current matching principles are reliable enough to reduce or minimize the risk of dnDSA formation.

Using the original HLA Matchmaker algorithm (54), Wiebe et al. (55) evaluated the development of *de novo* class-II DSAs in 286 kidney transplant recipients. Epitope mismatches were significantly more frequent in the patients who developed dnDSAs. In this study the optimal threshold for development of antibodies against HLA-DR was 10 mismatched epitopes and for HLA-DQ 17 mismatched epitopes (55). In a second study they investigated the interaction between medication adherence and degree of epitope mismatch. In this study in 596 renal recipients the optimal threshold for development of class II dnDSAs was 11 epitope mismatches for both HLA-DR and HLA-DQ. The combination of a high alloimmune risk (> 11 epitope mismatches) and tacrolimus trough levels below 5 ng/ml led to development of dnDSAs whereas patients with less than 11 epitope mismatches tolerated low tacrolimus trough levels (56). Recently they published the result of a study in 664 renal recipients. This study confirmed that the risk of dnDSAs was more strongly correlated to epitope mismatches as compared to conventional HLA mismatch. However, the threshold in this study was 7 epitope mismatches for HLA-DR and 9 for HLA-DQ (57). Also, Snanoudj et al. (58) investigated the epitope mismatch load by using HLA Matchmaker in 89 renal recipients. They found that epitope load was more strongly associated with dnDSAs compared to the number of serologic HLA mismatches. Of note, in this study the optimal threshold was 27 epitope mismatches (58).

So, one can easily appreciate potential pitfalls in these newer matching methods that were developed based on the epitope level. Although more accurate in predicting dnDSA development than conventional matching, defining a reliable threshold as a risk factor is difficult and needs to be solved. To identify patients at risk, or maybe equally relevant those with a lower risk and safer option to adjust immunosuppressive load, there is an urgent need for well-defined risk factors to guide clinical decision making.

## The Elderly and Age-Matching: the Eurotransplant Senior Program

Organ shortage and the continuously growing waiting list, demands a progressive expansion of the potential kidney donor pool. Therefore, boundaries of organ quality criteria are continuously stretched and more and more older donors with or without comorbid conditions are accepted for renal transplantation (8). With the acceptance of older donors, the proportion of what was historically called extended criteria donors (ECD) also increased significantly. Since 2015 donors in the US have been assessed by the so-called Kidney Donor Profile Index (KDPI) score, which is associated with the life expectancy of the graft. Kidneys with a KDPI > 85%, or high risk kidneys, are expected to function for more than 5.5 years and are therefore considered to be comparable to the previous so-called ECD kidneys (59).

It is well known that graft survival decreases with increasing donor age and decreasing organ quality, but also that the elderly still benefited from a successful kidney transplant using high risk kidneys in terms of life expectancy as compared to their waitlisted counterparts (60). Recipients of a high-risk kidney had a significantly lower mortality risk (RR 0.75; 95% CI 0.65-0.86), results confirmed by several studies (6, 60, 61).

It is widely accepted that each kidney should be allocated to the recipients in whom is it expected to survive the longest to improve the match between life expectancy of donor and recipient. Since older transplant recipients are more likely to die with a functioning graft and younger recipients have a higher chance on re-transplantation later in life, it seems logical to allocate older kidneys, with an increased chance of graft failure, to older recipients.

Therefore, in 1999 the Eurotransplant Senior Program (ESP) was implemented to shorten the waiting time for older transplant candidates and improve the perspective on patient survival with ESRD. In this program kidneys from donors > 65 years are allocated to recipients > 65 years with preferred local allocation in order to shorten cold ischemia times (CIT) and the likelihood of delayed graft function and/or rejection. To reach these goals, HLA matching was neglected, obviously resulting in a higher HLA mismatch rates in ‘old for old’ transplant programs. In 2008 the 5 years results were published and main goals were reached, waiting time decreased and CIT went down to 11.9 h compared to > 17 h in the regular ETKAS allocation program (62). However, there was a 5 to 10% higher rejection rate within ESP (29.1%) as compared to regular allocation. As mentioned in the study of Halleck et al. (33), this could be due to a higher HLA mismatch, especially HLA-DR, which led to significantly impaired graft survival. Indeed, in the ESP 92.9% of the recipients had 2 HLA-DR mismatches compared to 54.9% ≥ 1 HLA-DR mismatch in the normal allocation scheme.

## Changing the Strategy for Older Transplant Candidates

At the moment only a minority of selected elderly transplant candidates actually receive a kidney transplant and the mortality rate among this patient group is relatively high, especially the 1st year after transplantation or in case complications occur such as an acute rejection episode. In order to increase transplant rates, more older donors are accepted and preferably for older recipients, which in turn leads to more acute rejection episodes and rejection treatments.

In younger transplant recipients, the increased risk of acute rejection with the use of older donors could possibly be overcome with induction therapy or more potent maintenance therapy. In elderly the complex interplay between immunosuppression on the one hand and immune defense on the other hand is even more challenging due to pre-existing comorbidities, changes in pharmacokinetics of immunosuppressive drugs, polypharmacy and the immunosenescence mentioned earlier. Probably more balanced immunosuppressive protocols and more advanced immunological monitoring strategies are needed to balance this critical equipoise. A second, more feasible and practical strategy could be to change the allocation protocol to decrease the risk of acute rejection and/or DSA formation without the need of more clinical immunosuppression.

### Adjusting Maintenance Immunosuppressive Protocols

#### Calcineurin Inhibitors

Calcineurin inhibitors (CNI) remain the most potent immunosuppressive drugs in preventing acute rejection and have been critical to improve short-term graft survival. Due to the nephrotoxic potential of CNIs on long-term graft failure, there has been an overall shift toward reduction or CNI-withdrawal preferably later after transplantation. Since elderly are more susceptible to infections and the other side effects of immunosuppressive drugs and older kidney grafts are more vulnerable to CNI induced vasoconstriction and/or nephrotoxicity, several studies have suggested that especially elderly could benefit from CNI withdrawal or avoidance (63). In addition, the pharmacokinetics of CNIs change with increasing age and Staatz et al. (64) concluded in their review that especially maintenance therapy in older patients potentially needs more frequent monitoring and adjustments. Jacobson et al. (65) reported in a clinical trial that elderly (> 65 year) yielded similar trough levels with lower CNI dose and that dose-normalized trough levels were more than 50% higher in older patients.

Various studies have indicated that CNI-withdrawal in the regular population of renal transplant recipients may not be successful (66–71). The results of reduction of tacrolimus differ in literature, but the CTS study showed that graft survival is compromised below a trough level of 5 ng/ml and in those with high intra-patient variability (48). Several studies have reported an increase in dnDSA formation below a certain trough level. Gatault et al. (72) found only dnDSAs in the group with a mean tacrolimus trough level of 4.1 ng/ml. Recently Davis et al. (73) reported a 4-fold risk of dnDSAs for patients with a mean tacrolimus through level of 4-6 ng/ml as compared to ≥ 8 ng/ml in the 1st year after kidney transplantation. As mentioned before, the Winnipeg group addressed the risk of minimization of calcineurin inhibitors and the development of dnDSA in relation to epitope mismatch load. Both studies confirmed that patients with a higher epitope load were at risk for dnDSAs after minimizing immunosuppression (56, 58).

In elderly, Arbogast et al. (74) used an CNI free protocol after ATG induction followed by mycophenolate mofetil (MMF) and prednisone. Cumulative 5-year patient and allograft survival was 88 and 70%, respectively. And although these results are in itself excellent for older renal transplant recipients, the acute rejection rate was more than 25% and these patients returned to a regimen with a CNI after the rejection treatment which underlines the importance of a tailormade strategy rather than a standard protocol regarding immunosuppression (74).

#### mTOR Inhibitors

In order to reduce CNI exposure, also the mammalian target of rapamycin inhibitors (mTORi) have been introduced and positioned. Several randomized trials have been performed of which the most recent one is the large TRANSFORM study. In this study 2037 renal recipients were randomized to standard dose CNI + MMF or reduced CNI + mTORi. The latter proved to be non-inferior regarding a binary endpoint of BPAR or glomerular filtration rate (GFR) < 50ml/min/1.73m^2^. Benefits of the regimen with mTORi were a significantly reduced incidence of viral infections, which could be direct clinical benefit in the elderly. Although elderly were not excluded from this trial, mean age was 49.3 year (75). Recently the results from the SENATOR trial were reported. In this trial renal recipients participating in the Eurotransplant Senior Program (ESP) were included and 7-weeks after transplantation randomized to standard therapy with CNI + MMF or converted to MMF + everolimus and basiliximab at weeks 7 and 12. The patients who were converted and remained on everolimus had comparable kidney function and comparable rates of BPAR. Only 37.2% of the patients were actually randomized, identifying elderly as a vulnerable study population. From the patients who were randomized, 27.8% discontinued everolimus due to adverse events. This study underscores the challenge of randomized studies in elderly transplant recipients and general need for tailored treatment in this group.

### Allocation With Prospective HLA Matching

As expected, the ESP program achieved the goal to minimize cold ischemia time and also the anticipated reduction in the rate of delayed graft function. The higher incidence of acute rejection was not expected and this could suggest a greater role of immunogenicity in the context of less histocompatibility. One could overcome this increased rejection risk by increasing clinical immunosuppression. The elderly, however, already have a compromised immune system and are more vulnerable for infectious complications. In addition, marginal donors are more vulnerable for the nephrotoxic side effects of immunosuppressive drugs. Therefore, at least in theory, this may be a suitable strategy in a proportion of younger patients receiving older and more immunogenic kidneys but may not be the best options for the older transplant recipients.

A different strategy is to require a minimal degree of histocompatibility between donor and recipient while maintaining the shorter CIT and therefore the benefit of less delayed graft function. Due to a high degree of linkage between HLA-DR and HLA-DQ antigens, matching for HLA DR frequently results in matching on HLA-DQ (76). As previous studies proved, graft survival is worse even with 1 HLA-DR mismatch. Therefore, prospective HLA-DR matching with zero mismatches would be a potentially elegant strategy to improve rejection free survival without the need of excessive immunosuppression.

## Concluding Remarks

Given the inherent limited life expectancy of older patients, their best option when encountering ESRD would be the option of a kidney transplant as soon as possible. In order to reach this goal age matching is a suitable strategy and most patients will receive a kidney from an older deceased donor.

Even with a timely kidney transplant offer from an age-matched donor, there are other issues to consider in elderly recipients. Recipients of older kidneys are more susceptible to acute rejection with HLA class-II mismatch being a potentially preventable key risk factor also for the subsequent formation of DSAs. The mere fact that the older recipient has an older immune system as compared to adolescents, does not overcome the dominant effect of donor age over recipient age.

We therefore underlined the importance of prospective HLA matching in the allocation algorithm of older kidneys to older kidney transplant candidates. Since most DSAs are directed against HLA class II antigens, HLA-DR matching is likely to reduce the need for more intense clinical immunosuppression and/or additional acute rejection treatments, ensuing reduction of excess infectious cause morbidity and mortality while delivering the prospect of prolonged life expectancy.

To reintroduce prospective matching for HLA class-II antigens, the Eurotransplant Senior DR-compatible Program (ESDP) study was designed and the results will be important to guide future clinical practice.

## Author Contributions

GD wrote the first draft of the manuscript. JF wrote sections of the manuscript and revised the manuscript. Both authors contributed to the final manuscript revision, read and approved the submitted version.

## Conflict of Interest

The authors declare that the research was conducted in the absence of any commercial or financial relationships that could be construed as a potential conflict of interest.
